# Phase I study of a novel glioblastoma radiation therapy schedule exploiting cell-state plasticity

**DOI:** 10.1093/neuonc/noac253

**Published:** 2022-11-19

**Authors:** Jamie A Dean, Shyam K Tanguturi, Daniel Cagney, Kee-Young Shin, Gilbert Youssef, Ayal Aizer, Rifaquat Rahman, Lubna Hammoudeh, David Reardon, Eudocia Lee, Jorg Dietrich, Kaoru Tamura, Masaru Aoyagi, Lacey Wickersham, Patrick Y Wen, Paul Catalano, Daphne Haas-Kogan, Brian M Alexander, Franziska Michor

**Affiliations:** Department of Data Science, Dana-Farber Cancer Institute, Boston, Massachusetts, USA; Department of Stem Cell and Regenerative Biology, Harvard University, Cambridge, Massachusetts, USA; Department of Biostatistics, Harvard T.H. Chan School of Public Health, Boston, Massachusetts, USA; Department of Medical Physics and Biomedical Engineering, University College London, London, UK; UCL Cancer Institute, University College London, London, UK; Department of Radiation Oncology, Dana-Farber Cancer Institute, Harvard Medical School, Boston, Massachusetts, USA; Department of Radiation Oncology, Dana-Farber Cancer Institute, Harvard Medical School, Boston, Massachusetts, USA; Department of Data Science, Dana-Farber Cancer Institute, Boston, Massachusetts, USA; Department of Biostatistics, Harvard T.H. Chan School of Public Health, Boston, Massachusetts, USA; Center for Neuro-Oncology, Dana-Farber Cancer Institute, Harvard Medical School, Boston, Massachusetts, USA; Center for Neuro-Oncology, Massachusetts General Hospital, Harvard Medical School, Boston, Massachusetts, USA; Department of Radiation Oncology, Dana-Farber Cancer Institute, Harvard Medical School, Boston, Massachusetts, USA; Department of Radiation Oncology, Dana-Farber Cancer Institute, Harvard Medical School, Boston, Massachusetts, USA; Department of Radiation Oncology, Dana-Farber Cancer Institute, Harvard Medical School, Boston, Massachusetts, USA; Center for Neuro-Oncology, Dana-Farber Cancer Institute, Harvard Medical School, Boston, Massachusetts, USA; Center for Neuro-Oncology, Dana-Farber Cancer Institute, Harvard Medical School, Boston, Massachusetts, USA; Center for Neuro-Oncology, Massachusetts General Hospital, Harvard Medical School, Boston, Massachusetts, USA; Department of Neurosurgery, Tokyo Medical and Dental University, Tokyo, Japan; Department of Neurosurgery, Tokyo Medical and Dental University, Tokyo, Japan; Department of Radiation Oncology, Dana-Farber Cancer Institute, Harvard Medical School, Boston, Massachusetts, USA; Center for Neuro-Oncology, Dana-Farber Cancer Institute, Harvard Medical School, Boston, Massachusetts, USA; Department of Data Science, Dana-Farber Cancer Institute, Boston, Massachusetts, USA; Department of Biostatistics, Harvard T.H. Chan School of Public Health, Boston, Massachusetts, USA; Department of Radiation Oncology, Dana-Farber Cancer Institute, Harvard Medical School, Boston, Massachusetts, USA; Department of Radiation Oncology, Dana-Farber Cancer Institute, Harvard Medical School, Boston, Massachusetts, USA; Department of Data Science, Dana-Farber Cancer Institute, Boston, Massachusetts, USA; Department of Stem Cell and Regenerative Biology, Harvard University, Cambridge, Massachusetts, USA; Department of Biostatistics, Harvard T.H. Chan School of Public Health, Boston, Massachusetts, USA; The Broad Institute of MIT and Harvard, Cambridge, MA, USA; The Center for Cancer Evolution, Dana-Farber Cancer Institute, Boston, Massachusetts, USA; The Ludwig Center at Harvard, Boston, Massachusetts, USA

**Keywords:** cell-state plasticity, clinical trial, glioblastoma, mathematical modeling, radiation oncology

## Abstract

**Background:**

Glioblastomas comprise heterogeneous cell populations with dynamic, bidirectional plasticity between treatment-resistant stem-like and treatment-sensitive differentiated states, with treatment influencing this process. However, current treatment protocols do not account for this plasticity. Previously, we generated a mathematical model based on preclinical experiments to describe this process and optimize a radiation therapy fractionation schedule that substantially increased survival relative to standard fractionation in a murine glioblastoma model.

**Methods:**

We developed statistical models to predict the survival benefit of interventions to glioblastoma patients based on the corresponding survival benefit in the mouse model used in our preclinical study. We applied our mathematical model of glioblastoma radiation response to optimize a radiation therapy fractionation schedule for patients undergoing re-irradiation for glioblastoma and developed a first-in-human trial (NCT03557372) to assess the feasibility and safety of administering our schedule.

**Results:**

Our statistical modeling predicted that the hazard ratio when comparing our novel radiation schedule with a standard schedule would be 0.74. Our mathematical modeling suggested that a practical, near-optimal schedule for re-irradiation of recurrent glioblastoma patients was 3.96 Gy × 7 (1 fraction/day) followed by 1.0 Gy × 9 (3 fractions/day). Our optimized schedule was successfully administered to 14/14 (100%) patients.

**Conclusions:**

A novel radiation therapy schedule based on mathematical modeling of cell-state plasticity is feasible and safe to administer to glioblastoma patients.

**Key Points**

- A novel mathematical model-derived radiation therapy schedule improves survival in a mouse model.
- We translated this schedule from mice to glioblastoma patients using mathematical modeling.
- The novel schedule is feasible and safe to administer to glioblastoma patients.

**Importance of the Study**

Radiation therapy administration schedules fail to account for the dynamic cellular heterogeneity and plasticity of glioblastoma. We previously developed a mathematical model to quantitatively characterize this heterogeneity and plasticity, which we employed to optimize a radiation therapy schedule that substantially improved survival over a conventional schedule, in a mouse model of glioblastoma. In this study we adapted the schedule from mice to recurrent glioblastoma patients, using mathematical modeling, and performed a phase I trial to test the feasibility of administering our novel schedule. We successfully demonstrated that our novel schedule is indeed feasible and safe to administer, providing a route to a future efficacy trial. Hypothesis-generating analyses, comparing our trial with an external control dataset, are suggestive of a potential effect of the schedule on tumor response. We will investigate this finding in a future preclinical study.

Progress in developing new therapies for glioblastoma (GBM) has been slow.^1^ The addition of radiation therapy (RT) to surgery was shown to improve survival decades ago^2^ but since then, only temozolomide,^3^ lomustine-temozolomide,^4^ and tumor-treating fields^5^ have been shown to marginally improve survival. Furthermore, previous attempts to improve survival through RT dose/fractionation optimization based on the linear-quadratic model of classical radiation biology^6^ were largely unsuccessful.^7^ These approaches did not account for more recent data revealing that GBMs exhibit substantial intercellular heterogeneity^8^ and cell-state plasticity^9,10^ with variability in the radiation sensitivity of cell states.^11^

We hypothesized that these advances in the understanding of disease biology, together with improvements in mouse modeling,^12^ could be exploited to develop a new mathematical model of GBM radiation response that can inform superior administration schedules. We previously demonstrated that such a model could be used to optimize a radiation administration schedule that substantially improved survival over standard schedules in a genetically engineered mouse model of GBM.^13^ The survival advantage was equivalent to doubling the efficacy of each Gray of radiation administered using the standard schedule. The prolonged survival achieved by the optimized administration schedule was proposed to be due to increasing the fraction of glioma stem-like cells.^13^ These cells, while more radiation resistant, proliferate more slowly following irradiation, thereby increasing the time to recurrence.

To investigate the clinical potential of a model-informed optimized schedule, we used mathematical modeling to adapt the approach from mice to humans, and conducted a phase I trial, assessing the feasibility and safety of administering the schedule.

## Materials and Methods

### Translating the Schedule From Mice to Humans Using Mathematical Modeling

We used our mathematical model, described in Leder *et al.*,^13^ to simulate and compare different administration schedules. We constrained the maximum number of fractions to be administered on a single day to be 3 and the overall treatment time to be 10 weekdays. We set the schedule start day to be Monday. Since we cannot be certain that the optimum interfraction interval to achieve cell-state transitions from the rapidly proliferating to slowly proliferating states is independent of dose, we constrained the dose per fraction to be 1.0 Gy (ie, the dose per fraction used in the optimized schedule in our preclinical study) on days on which multiple fractions are administered.

We performed toxicity modeling using the biologically effective dose formalism based on the linear-quadratic model of radiation dose response.^6^ The biologically effective dose, BED, is given by the expression.


$$\begin{array}{l} \begin{array}{l} BED=nd\left(1+\frac{d}{\alpha /\beta }\right) \end{array} \end{array}$$ (1)

where *n* is the number of fractions, *d* is the dose per fraction, and the α/β ratio is a tissue-specific radiation fraction size sensitivity factor, the values of which are based on preclinical and clinical data. We used α/β=2 Gy, which is the commonly used value for tissues of the central nervous system and recommended for use in the setting of brain re-irradiation.^14^ When different fractionation schemes are combined in a single course of treatment, the total biologically effective dose for the combined phases, BED_total_, is the sum of the biologically effective doses of the *P* individual phases,


$$\begin{array}{l} \begin{array}{l} BED_{total}=\underset{i=1}{\overset{P}{\sum }}\;BED_{i} \end{array} \end{array}$$ (2)

We constrained *P* to have a maximum value of 2 as higher values would require substantial additional work for radiation oncology departments to administer and would likely not lead to a significant survival advantage. We optimized the control parameters *n*, *d*, and *P*, to obtain a schedule for evaluation in patients.

We performed sensitivity analyses by using the model to simulate the impact of different interfraction intervals and starting the schedule on different days of the week on the predicted tumor response dynamics. Code for our analyses is available at https://github.com/jamiedean/glioblastoma-radiation-therapy-schedule.

### Phase I Trial to Assess Feasibility of Administering the Novel Radiation Therapy Schedule

Recurrent GBM patients (based on contemporary pathologic grading systems) who were previously treated with definitive neurosurgical biopsy or resection followed by RT with or without systemic therapy, deemed appropriate for re-irradiation, at least 18 years old and with a Karnofsky Performance Status of at least 70 were eligible for this feasibility study, which was approved by the Dana-Farber Cancer Institute Institutional Review Board (DF 18-105; NCT03557372). Exclusion criteria were either more than one prior course of RT to the local site of progressive disease, RT to the local site of progressive disease within 3 months of the anticipated start of re-irradiation, brainstem or optic structures involvement, and no radiologically definable tumor cavity. There were no limits on the number of prior relapses or use of prior therapies (other than the stated exclusion criteria relating to prior RT), including bevacizumab. Written informed consent was received from all patients prior to participation. Subjects were permitted to receive bevacizumab, but not other therapies, concurrent with re-irradiation. All subjects enrolled underwent a CT simulation with rigid stereotactic immobilization using the QFix Encompass™ SRS Fibreplast® System. The Gross Tumor Volume was defined using CT and contrast-enhanced MRI obtained prior to the initiation of re-irradiation. In postsurgical cases where no residual enhancing tumor was noted, the postoperative resection cavity was contoured as the Gross Tumor Volume. Treatment of subclinical disease through a Clinical Target Volume was not performed in any patient treated given the re-treatment setting of this cohort, though the protocol did allow an optional Clinical Target Volume expansion of up to 5 mm for lesions <3.5 cm in maximal diameter or for lesions not previously irradiated. A Planning Target Volume (PTV) was generated using a margin of 2–3 mm with daily pretreatment kV and cone-beam CT imaging to confirm setup accuracy.

External beam radiation treatment plans were optimized using inverse planning (Varian Eclipse Version 15.6) using the Volumetric Arc Therapy technique. Sequential plans were generated to deliver a schedule of 7 fractions of 3.96 Gy (QD) followed by 9 fractions of 1.0 Gy (TID) based on pre-defined coverage (Supplementary Table 6) and normal-tissue (Supplementary Table 7) dosimetry objectives. Treatment was permitted to begin on any weekday based on the previously described sensitivity analysis, which predicted a similar benefit of the novel schedule over the standard re-irradiation schedule, irrespective of which day of the week treatment started (Supplementary Figure 2D).

The primary endpoint was the successful completion of schedule administration, defined as administering the TID fractions within 1 h of the prescribed times and the QD fractions within 24 h of the prescribed times. These time windows for schedule adherence were based on sensitivity analyses (Supplementary Figure 2C–D). The final target accrual for the study, based on the following power calculation, was 14 cases. If 13 out of 14 patients (86%) were able to complete the proposed schedule, this would demonstrate that the one-sided 95% upper limit (exact binomial distribution) for the population non-adherence is 30%. This non-adherence rate, or lower, would be deemed acceptable to progress to a subsequent efficacy trial. If all patients were able to complete the proposed schedule, the study would demonstrate that the one-sided 95% upper confidence interval for the population non-adherence is no higher than 20%. Of relevance, a recent trial attempted to deliver 90 TID fractions of radiation therapy to newly diagnosed GBM patients.^15^ The schedule was successfully administered in 22/27 (81%) patients who started radiation therapy. The one-sided 95% upper limit (exact binomial distribution) for the population non-adherence was, therefore, 35%. Based on this adherence rate, delivery of the schedule was deemed feasible.

Subjects were assessed with a physical and neurologic examination, toxicity assessments (Common Terminology Criteria for Adverse Events (CTCAE) v4.0), and patient-reported outcome measures (MDASI-BT: M. D. Anderson Symptom Inventory—Brain Tumor survey) at baseline and at 4-weeks post-treatment. Routine MRI surveillance imaging, toxicity assessment, and subsequent clinical follow-up were performed in conjunction with Radiation Oncology and Neuro-Oncology treatment teams every 1–3 months per standard of care. Radiographic response to treatment was assessed using a combination of standard MRI, advanced imaging (diffusion-weighted imaging, perfusion), and clinical assessment using the Response Assessment in Neuro-Oncology (RANO) working group methodology.^16^ Progression-free and overall survival were estimated using Kaplan-Meier analysis.

### Comparison of Survival and Recurrence Pattern With an External Control Arm

An external control arm was generated by collecting available data for recurrent GBM patients treated with re-irradiation at our institution (92 courses of re-irradiation). We tested the associations between our novel administration schedule and both progression-free and overall survival (*n* = 92), and distant versus local progression (*n* = 73; data not available for all patients) by performing multivariable Cox proportional hazards regression modeling and logistic regression, respectively. Conducting covariate adjustment in this manner has been shown to be equivalent or more reliable than causal inference methods, such as propensity scoring, in the context of small sample sizes.^17^ In an alternative analysis designed to use each patient as their own internal control,^18^ we calculated the ratios of times to progression following re-irradiation and both the prior line of therapy and first-line therapy for each patient. We compared these ratios between the trial and external control patients using *t*-tests.

The pattern of progression was defined as the location of the earliest progression following re-irradiation. For the study cohort, all follow-up imaging studies were fused volumetrically with baseline and subsequent imaging studies. Local progression was defined by progressive enhancing within or abutting the PTV or by progressive infiltrating non-enhancing disease; distant progression was defined by non-continuous disease which did not abut the treated PTV; marginal progression was defined by distant progressive disease occurring within 1 cm of the PTV^19^ with otherwise stable disease locally. Where local and distant progressions were detected simultaneously, these were coded as local progressions. Patients who died without progressions being detected on imaging were censored from analyses of pattern of progression (*n* = 2). For the external control arm, progression was determined in the absence of radiation treatment fields/DICOM images, for which local progression was defined by any progressive change within 1–2 cm of the initial enhancing/post-surgical volume or within previous FLAIR hyperintense non-enhancing disease; all other progression was considered distant. As progressions were only coded as local or distant and not marginal in the external control arm, when comparing the trial and control patients we performed two separate logistic regression analyses, with marginal progressions in the trial coded as either local or distant. In addition to treatment with our experimental schedule, we included the following covariates: sex, age, Karnofsky performance status, tumor size (product of two perpendicular tumor diameter measurements in millimeters), progression number, RT biologically effective dose (calculated using equation (1) with α/β   =10 Gy) and bevacizumab treatment (prior or concurrent). Karnofsky performance status data were unavailable for 9 patients in the external control cohort. These missing data were imputed using the median value for the cohort.

## Results

### Mathematical Modeling Enables the Derivation of a Novel Radiation Therapy Schedule Predicted to Increase Survival of Recurrent GBM Patients

Before translating our novel radiation schedule from mice to humans, we first predicted its benefit to GBM patients by developing linear regression models to predict the magnitude of GBM patient survival benefits afforded by interventions based on their benefit in the mouse model employed in our prior study (Appendix 1). Our regression modeling predicted a hazard ratio for the benefit of the novel schedule over a conventional schedule of 0.74. We, therefore, deemed that the predicted clinical benefit of our novel schedule was sufficient to warrant clinical investigation.

Standard-of-care therapy for newly diagnosed GBM is surgery followed by radiation therapy with concurrent and adjuvant temozolomide.^3^ To investigate how to optimally administer radiation together with temozolomide, we recently developed a mathematical model of combination treatment, finding that the optimal radiation therapy schedule is different when administered together with temozolomide as compared to radiation monotherapy.^20^ Therefore, our original model^13^ is not suitable for designing novel combination treatment schedules. However, patients commonly receive radiation therapy alone for the treatment of recurrent GBM. We therefore focused on this setting for the clinical translation of our mathematical model-based radiation schedule.

To design a clinical study, we used our previously developed mathematical model^13^ to translate the optimized schedule used in mouse experiments (based on delivering 10 Gy over 5 days) to humans. There is no standard-of-care radiation therapy administration schedule for re-irradiation of recurrent GBM in humans. However, a commonly used schedule in routine practice^21^ and clinical trials^22^ (NCT02709226, NCT02025231) is 35 Gy administered over 10 daily fractions. We therefore used this schedule as the basis for designing a new regimen suitable for the treatment of recurrent GBM patients. To ensure equivalence in terms of predicted side effects between our novel schedule and the standard schedule, we performed toxicity modeling based on the commonly used linear-quadratic model,^6^ with , α/β=2 Gy,^14^ to constrain the allowable administration schedules (Methods). We also required our new schedule to have the same or shorter overall treatment times (2 weeks) as the standard schedule, no weekend administrations, and a maximum of 3 administrations per day, being mindful of patient-centered approaches.

The details of our mathematical model used to design the schedule have been previously described.^13^ Briefly, the model quantifies the proliferation and radiation response of a treatment-resistant stem-like cell population and a treatment-sensitive differentiated cell population. These two populations are coupled by differentiation and radiation-induced dedifferentiation processes, with the rate of dedifferentiation depending on the time interval between successive fractions of radiation. Maximal dedifferentiation was inferred, using mouse data, to occur with a 3.25 h time interval between radiation fractions. Our preclinical study uncovered that administering radiation with a 3–4 h interval between fractions substantially increased survival compared with administering the same total dose of radiation using multiple different alternative schedules. Our mathematical model, validated in the mouse model, suggests that the reason for this survival improvement is due to increasing the fraction of stem-like cells, which are more resistant to radiation than differentiated cells but undergo a longer period of quiescence before beginning to proliferate again, thereby delaying the time to tumor progression.

Before using this model to design a human schedule, we used available data to explore whether the 3.25 h interval between fractions associated with improved survival in the mouse model was likely to be consistent between species (Appendix 2). The evidence suggested that the efficacy of using this time interval may be consistent across species; we therefore used the same time-dependence for the rate of dedifferentiation when designing the human schedule. Furthermore, we were cautious about deliberately enriching tumors for treatment-resistant stem-like cells so before commencing our study we used existing data to explore the association between glioma stem-like cell dynamics and survival in patients (Appendix 3). From these analyses, we concluded that our strategy was unlikely to worsen patient survival.

**Figure 1.**
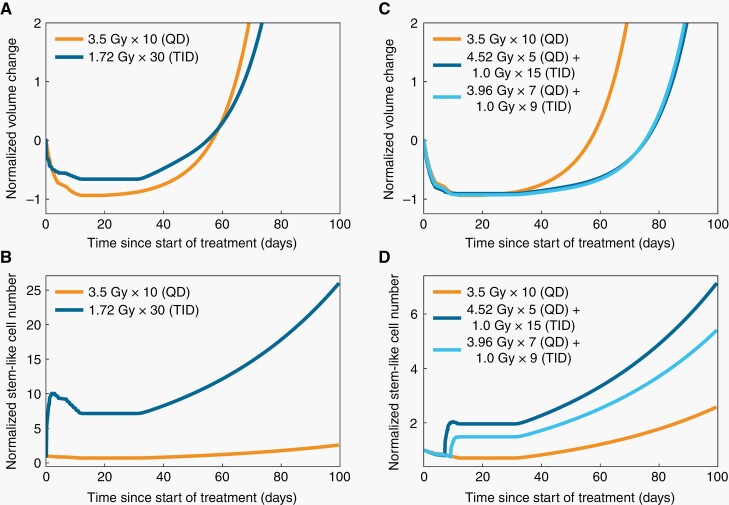
Mathematical modeling identifies a radiation therapy administration schedule predicted to improve survival of recurrent GBM patients. (A) Predicted tumor volume dynamics resulting from a radiation schedule of 51.6 Gy in 30 fractions with three times daily dosing with 3.25 h interfraction intervals compared to those from a schedule of 35 Gy in 10 fractions. (B) Predicted stem-like cell dynamics resulting from the administration schedules in (A). (C) Predicted effect of different two-phase radiation administration schedules on tumor volume dynamics. (D) Predicted stem-like cell dynamics resulting from the administration schedules in (C). QD—once daily dosing; TID—three times daily dosing.

While our preclinical study suggested that employing a 3.25 h interfraction interval prolongs survival,^13^ model simulation of 2 weeks of three times daily dosing (TID) with a 3.25 h interfraction interval (relaxing the 1.0 Gy per fraction constraint to enable equal biologically effective doses) predicts that this strategy would only be marginally beneficial (Figure 1A). By visualizing the predicted dynamics of the stem-like cell compartment (Figure 1B), we hypothesized that this finding is due to increasing the fraction of radioresistant stem-like cells early during the treatment course, reducing the effectiveness of the remaining fractions of radiation. When simulating alternative treatment strategies, we found that survival is optimized within the constraints by an initial phase of hypofractionation followed by a TID administration phase, with a 3.25 h interfraction interval. The predicted optimal treatment schedule is 5 fractions of 4.52 Gy (QD) followed by 15 fractions of 1.0 Gy (TID; 3.25 h interfraction interval; Figure 1C). The predicted cell population dynamics indicate that this strategy produces a balance between initially minimizing the total cell number—with hypofractionation—and then maximizing the stem-like cell fraction at the end of treatment—with the TID administrations (Figure 1D). The model predicts that this schedule results in a cytoreduced tumor comprised mainly of slowly proliferating stem-like cells at the end of treatment (Supplementary Figure 2A).

As TID administrations are less convenient than QD administrations, we tested whether schedules with fewer TID administrations could achieve close to the predicted benefit achieved by the optimal schedule. Our simulations revealed that a schedule of 7 fractions of 3.96 Gy (QD) followed by 9 fractions of 1.0 Gy (TID; 3.25 h interfraction interval) is predicted to be minimally inferior to the optimal schedule (Figure 1C–D). We therefore selected this schedule for evaluation in GBM patients. We envisioned that a 1 h window around the prescribed 3.25 h interfraction interval would be necessary to ensure that the schedule was feasible to administer. To investigate how this time window would affect the predicted survival benefit, we simulated treatments with varying interfraction intervals (Supplementary Figure 2B). We found that a 1 h window on either side of the prescribed 3.25 h interval did not compromise the predicted efficacy excessively, and that treatments within this window remained superior to the predicted efficacy of the standard schedule. We therefore, reasoned that this interval provided a reasonable balance between predicted efficacy and practicality. We found that for the daily treatments, the time of day at which radiation was administered did not substantially affect the predicted tumor response dynamics (Supplementary Figure 2C). We also found that the day of the 5-day work week that radiation therapy was started was not predicted to meaningfully affect treatment response (Supplementary Figure 2D).

### The Novel Radiation Therapy Schedule is Feasible and Safe to Administer to Patients

We next sought to establish whether this nonstandard treatment approach could be feasibly and safely administered to patients with recurrent GBM. To address this question, we designed and completed a prospective pilot study to evaluate the feasibility and toxicity of our novel radiation administration schedule: 7 fractions of 3.96 Gy (QD) followed by 9 fractions of 1.0 Gy (TID; 3.25 h interfraction interval (Methods). We enrolled recurrent glioblastoma patients who were previously treated with definitive neurosurgical biopsy or resection followed by RT, deemed appropriate for re-irradiation; the inclusion and exclusion criteria are provided in Methods. The primary outcome was feasibility, defined as the successful completion of radiation therapy for at least 13 of 14 patients (power calculation described in Methods). Successful completion of radiation was defined as receipt of all scheduled fractions of daily administrations on the same day as the prescribed administration and within 1 h of TID administrations, based on our analysis of the effects of varying the interfraction intervals (Supplementary Figure 2B–C).

Patient characteristics are described in Table 1. The median age at the time of re-irradiation was 54 years (range 23–70 years), the median time from the initial diagnosis to re-irradiation was 10.5 months (range 5.8–55.5 months), the median number of recurrences was 2 (range 1–3), and the median number of prior lines of therapy for recurrence was 1 (range 1–3). Most patients had large tumors (median volume 94.0 cc; comparison with an external control cohort in Supplementary Figure 3A) and had previously received bevacizumab (78.6%), indicating a poor prognosis cohort. Molecular profiles similarly indicated a poor-risk biological cohort including predominance of IDH-1 wild type (85.7%), MGMT unmethylated (57.1%) tumors with EGFR, PTEN, and CDKN2A profiles in keeping with molecular glioblastoma (Table 1). The schedule was successfully administered to 14/14 (100%) patients without any delays or dose modifications (Table 2). Thus, our primary endpoint was met.

**Table 1.** Characteristics of Patients in the Clinical Trial

Variable	*N* = 14
Age (years)	
Median (range)	53.6 (23.2–70.3)
Mean (sd)	51.6 (14.5)
Sex	
Female	4 (28.6%)
Male	10 (71.4%)
Race	
White	14 (100%)
Black or African American	0 (0%)
Asian	0 (0%)
American Indian or Alaskan Native	0 (0%)
More than one race	0 (0%)
Other	0 (0%)
KPS	
90	7 (50.0%)
80	4 (28.6%)
70	3 (21.4%)
De-novo vs. Secondary	
De-novo	14 (100%)
Secondary	0 (0%)
IDH-1 status	
Wild-type	12 (85.7%)
Mutant	2 (14.3%)
MGMT promoter status	
Methylated	6 (42.9%)
Unmethylated	8 (57.1%)
EGFR status	
Wild-type	2 (14.3%)
Gain	7 (50%)
Mutant	1 (7.1%)
Gain and mutant	4 (28.6%)
PTEN status	
Wild-type	2 (14.3%)
1–2 copy loss	8 (57.1%)
Mutant	3 (21.4%)
Unknown	1 (7.1%)
CDKN2A status	
Copy-neutral	4 (28.6%)
1–2 copy loss	8 (57.1%)
Gain	1 (7.1%)
Mutant	1 (7.1%)
Taking steroids at time of registration	
No	6 (42.9%)
Yes	7 (50.0%)
Unknown	1 (7.1%)
Number of relapses (including present)	
1	2 (14.3%)
2	8 (57.1%)
3	4 (28.6%)
Number of prior therapies for relapse	
1	8 (57.1%)
2	5 (35.7%)
3	1 (7.1%)
History of prior treatment with temozolomide	
No	1 (7.1%)
Yes	13 (92.9%)
History of prior treatment with bevacizumab	
No	3 (21.4%)
Yes	11 (78.6%)
History of prior treatment with immunotherapy	
No	8 (57.1%)
Yes	6 (42.9%)
History of prior clinical trial systemic treatment for GBM	
No	3 (21.4%)
Yes	10 (71.4%)
Unknown	1 (7.1%)
Other prior systemic therapy(ies)	
No	5 (35.7%)
Yes	9 (64.3%)
History of prior treatment with Novo-TTF	
No	11 (78.6%)
Yes	2 (14.3%)
Unknown	1 (7.1%)
Other interventional treatment(s)	
No	6 (42.9%)
Yes	8 (57.1%)
History of neurological deficits	
No	6 (42.9%)
Yes	8 (57.1%)
History of seizures	
No	3 (21.4%)
Yes	10 (71.4%)
Unknown	1 (7.1%)
Were the seizures controlled prior to study registration	
No	0 (0%)
Yes	10 (64.3%)
Not applicable	4 (35.7%)
History of other non-CNS malignancy	
No	13 (92.9%)
Yes	1 (7.1%)
Was non-CNS malignancy controlled or in remission at time of study registration	
No	1 (7.1%)
Yes	0 (0%)
Not applicable	13 (92.9%)
History of radiation necrosis	
No	12 (85.7%)
Yes	2 (14.3%)
Other relevant prior medical conditions	
No	10 (71.4%)
Yes	4 (28.6%)

**Table 2.** Treatment Related Information

Time from initial diagnosis to first recurrence diagnosis (months)	
Median (range)	10.5 (5.8–55.5)
Mean (sd)	15.4 (12.8)
Time from initial diagnosis to re-irradiation (months)	
Median (range)	14.8 (0.8–60.7)
Mean (sd)	18.5 (13.7)
Planning Target Volume (cc)	
Median (range)	94.0 (28.8–318.4)
Mean (sd)	114.0 (83.2)
Concurrent bevacizumab	
No	3 (21.4%)
Yes	11 (78.6%)
Completion of radiation therapy	
Completed	14 (100%)
Not completed	0 (0%)
Dose modifications/delays	0 (0%)

The toxicity and patient-reported outcomes are described in Tables 3 and 4, respectively. Not all toxicity and patient-reported outcome data were collected (Supplementary Table 5) due to incomplete adherence to protocol follow-up related to disease progression and/or patient preference and challenges by patients to complete electronic questionnaires. There were four Grade 3 or higher toxicity events, including one patient with Grade 3 headache, one patient with Grade 4 hydrocephalus (attributed to out-of-field to disease progression) and one patient with Grade 4 cerebral edema and seizures (likely attributable to disease progression and/or treatment). Patient-reported outcome data showed few high-grade complaints, primarily related to mood, cognitive function, appearance, and energy.

**Table 3.** All Grade Adverse Events Regardless of Attribution at Baseline and Post-Baseline (CTCAE version 5.0)

		Baseline Grade		Post-Baseline Grade				
		1 (*n*)	2 (*n*)	1 (*n*)	2 (*n*)	3 (*n*)	4 (*n*)	5 (*n*)
**Category**		1	.	.	.	.	.	.
Ear and labyrinth disorders	Tinnitus							
Gastrointestinal disorders	Nausea	3	.	1	.	.	.	.
General disorders and administration site conditions	Death NOS	.	.	.	.	.	.	7
	Disease progression	.	.	.	.	.	.	1
	Fatigue	8	3	2	.	.	.	.
	Gait disturbance	1	.	.	.	.	.	.
	General disorders and administration site conditions— Other, specify	1	1	.	.	.	.	.
Infections and infestations	Infections and infestations—Other, specify	.	.	.	1	.	.	.
Metabolism and nutrition disorders	Anorexia	2	.	.	.	.	.	.
Musculoskeletal and connective tissue disorders	Arthralgia	1	.	.	.	.	.	.
	Back pain	2	.	.	.	.	.	.
	Generalized muscle weakness	.	2	.	1	.	.	.
	Muscle weakness lower limb	.	.	1	.	.	.	.
	Muscle weakness upper limb	1	.	.	.	.	.	.
Nervous system disorders	Ataxia	1	.	1	.	.	.	.
	Cognitive disturbance	1	.	.	.	.	.	.
	Concentration impairment	1	.	.	.	.	.	.
	Dysarthria	1	2	.	.	.	.	.
	Dysphasia	1	.	.	.	.	.	.
	Edema cerebral	.	2	1	.	.	1	.
	Headache	7	.	.	.	1	.	.
	Hydrocephalus	.	.	.	.	.	1	.
	Memory impairment	1	.	.	.	.	.	.
	Muscle weakness right-sided	.	2	.	.	.	.	.
	Seizure	6	3	.	.	.	1	.
Psychiatric disorders	Anxiety	1	.	.	.	.	.	.
	Confusion	1	.	.	.	.	.	.
	Depression	1	.	.	.	.	.	.
Maximum grade of any toxicity		8	6	2	1	0	0	8

**Table 4.** Number of Patients Experiencing Patient-Reported Symptoms and their Impact on Quality of Life in the Clinical Trial (Assessed Using MDASI-BT)

Patient-reported outcome	Grade at Baseline (*N* = 8)*											Grade at 4 Weeks Following Treatment (*N* = 6)*										
	0	1	2	3	4	5	6	7	8	9	10	0	1	2	3	4	5	6	7	8	9	10
Pain	5	1	0	0	0	0	1	0	0	0	0	3	0	1	0	0	1	0	0	0	0	0
Fatigue (tiredness)	1	0	2	0	2	1	0	0	2	0	0	1	0	1	0	2	1	0	0	0	0	1
Nausea	6	0	1	0	1	0	0	0	0	0	0	3	1	0	1	0	0	0	0	0	0	0
Disturbed sleep	3	0	0	2	1	0	0	0	0	0	1	3	0	1	2	0	0	0	0	0	0	0
Distressed (upset)	3	0	0	1	2	1	0	0	1	0	0	1	0	1	1	0	0	0	1	1	0	0
Shortness of breath	6	1	1	0	0	0	0	0	0	0	0	4	0	1	0	0	0	0	0	0	0	0
Remembering things	2	0	2	2	0	0	1	1	0	0	0	3	0	0	0	0	0	1	0	0	1	0
Lack of appetite	4	0	0	1	0	2	1	0	0	0	0	3	0	0	1	0	0	0	1	0	1	0
Drowsy (sleepy)	3	0	1	1	0	1	1	0	1	0	0	2	0	0	1	0	1	0	0	0	0	1
Dry mouth	4	0	2	0	1	1	0	0	0	0	0	5	0	0	0	1	0	0	0	0	0	0
Sad	3	0	2	1	1	0	0	1	0	0	0	2	0	0	1	0	0	0	1	1	0	0
Vomiting	7	1	0	0	0	0	0	0	0	0	0	5	0	1	0	0	0	0	0	0	0	0
Numbness or tingling	5	1	0	0	1	0	0	1	0	0	0	3	0	1	0	0	0	0	0	1	0	0
Weakness	4	0	0	3	0	0	0	1	0	0	0	3	0	0	0	2	0	1	0	0	0	0
Understanding	5	0	1	0	1	0	0	1	0	0	0	3	0	1	0	0	0	0	0	0	1	0
Speaking	4	0	0	0	1	1	0	1	0	0	0	2	1	0	1	1	1	0	0	0	0	0
Seizures	8	0	0	0	0	0	0	0	0	0	0	5	0	0	0	0	0	0	0	0	0	0
Concentrating	4	0	1	1	0	1	0	0	1	0	0	2	1	0	0	1	0	0	0	1	0	0
Vision	5	0	0	0	1	0	1	1	0	0	0	2	1	1	0	0	0	0	1	0	0	0
Appearance	7	0	0	0	0	0	0	0	1	0	0	2	0	0	0	0	1	0	1	1	0	0
Bowel pattern (diarrhea or constipation)	6	0	0	1	0	0	0	0	1	0	0	3	0	0	0	0	0	0	0	1	0	0
Irritability	5	1	0	0	1	0	0	1	0	0	0	2	0	1	0	1	2	0	0	0	0	0
General activity	2	2	1	0	2	1	0	0	0	0	0	2	0	0	0	0	0	1	0	0	0	1
Mood	3	0	0	0	3	2	0	0	0	0	0	1	0	0	3	0	0	0	2	0	0	0
Work (including work around the house)	1	0	0	1	1	2	0	1	0	1	1	2	0	0	0	0	0	0	1	0	0	1
Relations with other people	3	0	2	0	1	0	2	0	0	0	0	2	0	1	1	0	0	0	1	1	0	0
Walking	3	0	0	1	2	0	0	1	1	0	0	2	0	0	0	0	1	0	1	0	1	0
Enjoyment of life	2	1	1	0	1	0	0	1	1	1	0	0	0	0	2	2	0	0	0	0	1	1

*Not all patients completed MDASI-BT, therefore the data from the two timepoints may be from different patients.

**Figure 2.**
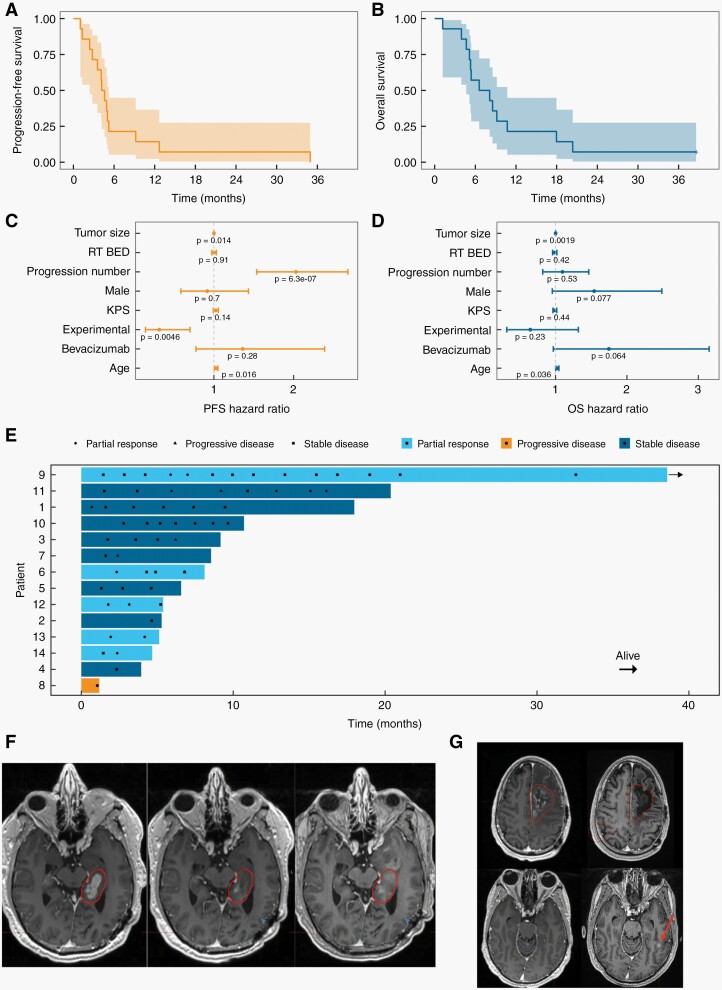
Patterns of tumor response and survival of patients treated in the clinical trial. (A) Kaplan-Meier plot for progression-free survival. (B) Kaplan-Meier plot for overall survival. (C) Cox proportional hazards regression model hazard ratios for progression-free survival. (D) Cox proportional hazards regression model hazard ratios for overall survival. (E) Swimmer plot of tumor response. The points indicate the responses at individual time points and the colors indicate the best response for each patient. (F) MRI scans of Patient 6 at the time of radiation therapy planning (left), 3 month follow-up (center) and 6 month follow-up with progression (right) showing a representative example of stable to regressed disease locally within the Planning Target Volume (contour line) but evidence of progressive enhancing, hypercellular disease with elevated cerebral blood volume immediately anterior to the Planning Target Volume. The patient died of disease 5 weeks after this time point. (G) MRI scans of Patient 1 at the time of radiotherapy planning (left) and 1 month follow-up (right) showing an example of a distant progression outside of the radiation therapy field (arrow), with stable to well controlled disease locally within the Planning Target Volume (contour line).

Median progression-free survival and overall survival were 4.4 months (95% CI 2.4–5.2 months) (Figure 2A), and 7.3 months (95% CI 4.7–10.7 months) (Fig. 2B), respectively. Comparison of progression-free and overall survival to an external control arm of recurrent GBM patients treated with re-irradiation (*n* = 92; Supplementary Table 8), adjusting for differences in known prognostic factors (the distributions of which are shown in Supplementary Figure 3A) using multivariable Cox proportional hazards regression models, demonstrated a significant effect of our schedule on progression-free (HR = 0.32; *P* = .0046; Figure 2C), but not overall survival (HR = 0.59; *P* = .15; Figure 2D). The ratios of time to progression following re-irradiation to time to progression following either the previous line of therapy (*P* = .011; Supplementary Figure 3B) or first-line therapy (*P* = .011; Supplementary Figure 3C) were significantly higher for the trial patients than the external control patients. Objective response rates were 36%, and 93% for patients with partial response or stable disease, respectively (Figure 2E).

Notably, of the 12 patients with pattern of progression data available, 2 patients (17%) had local recurrences and 10 patients (83%) had out-of-field failures. Of these, 5 (42%) were marginal (Figure 2F) and 5 (42%) were distant (Figure 2G). Progression was determined using a combination of advanced imaging (diffusion-weighted imaging, perfusion) and clinical assessment. In contrast, the external control arm had a distant failure rate of only 16%. Adjusting for differences in known prognostic factors between the 2 cohorts (the distributions of which are shown in Supplementary Figure 3A) using a multivariable logistic regression model indicated a significant relationship between our schedule and an increased rate of distant failure (marginal progression treated as local: OR = 6.1; *P* = .036; marginal progression treated as distant: OR = 44.8; *P* = .00052; Supplementary Figure 3D–E).

## Discussion

Given the relatively low success rate of novel interventions in oncology,^23^ we believe that attempting to improve the efficacy of existing standard-of-care therapies represents an under-utilized opportunity. A promising approach to enhance standard-of-care therapies is optimizing their administration schedules based on a quantitative understanding of the biology of treatment responses.^24^ This approach warrants increased attention for several reasons: (i) prior demonstration of clinical benefit renders the approach less likely to prove futile; (ii) few if any studies explore alternate administration schedules based on rational modeling, and (iii) the novel regimen has added advantages of being simple to implement, cost-effective, and often covered by health insurance plans. We applied this approach to the treatment of GBM—heterogeneous, treatment-resistant tumors for which the vast majority of novel interventions have failed to improve survival.^1^ We focused on improving the efficacy of radiation therapy, a standard-of-care therapy used in the treatment of most GBM patients.

In an initial study, we employed a combination of mathematical and mouse modeling to quantitatively characterize the dynamic heterogeneity and plasticity of GBM radiation responses. We exploited this quantitative understanding to optimize a radiation schedule, which substantially improved survival compared with a standard schedule in a preclinical trial using mouse models.^13^ Here we translated our preclinical treatment strategy from the laboratory to glioblastoma patients (Figure 1, Appendices 1–3). We used mathematical modeling to design a new schedule optimized for the treatment of patients, accounting for the different total dose and overall treatment time used in the re-irradiation of recurrent GBM patients as compared with mouse radiation therapy and incorporating toxicity modeling (Figure 1). Our model identified a regimen of 7 fractions of 3.96 Gy (QD) followed by 9 fractions of 1.0 Gy (TID; 3.25-h interfraction interval) predicted to confer a survival advantage over conventionally fractionated schema, perhaps by achieving a balance between initial cytoreduction with hypofractionated radiation therapy and maximizing the stem cell-like fraction through TID dosing. We completed a clinical trial that successfully demonstrated that this novel treatment approach is feasible and safe to administer to recurrent GBM patients (Figure 2, Tables 1–4). Beyond the specific intervention that we translated from the laboratory to the clinic, our study provides a template that can be used by others when deciding whether to translate apparently promising preclinical results into clinical trials (Appendix 4).

The progression-free (4.4 months) and overall (7.3 months) survival of patients in our clinical trial were similar to those observed in studies of re-irradiation of recurrent GBM treated with conventional schedules.^25,26^ However, differences in pre and post-protocol therapies and potential selection biases limit the value of such comparisons. Of note, most of the patients in our trial had large tumors that were pretreated, particularly with bevacizumab, an agent typically reserved for large and symptomatic tumors at our institution (Tables 1–2, Supplementary Figure 3A). We then performed hypothesis-generating efficacy analyses using an external control arm of recurrent GBM patients treated with conventionally employed re-irradiation schedules. Multivariable survival analyses including known prognostic factors showed a significant association between our novel schedule and progression-free survival (Figure 2C), but not overall survival (Figure 2D). The ratios of time to progression following re-irradiation to time to progression following either the previous line of therapy or first-line therapy (Supplementary Figure 3B–C) were significantly higher in our trial than the external control cohort. Patients in our trial exhibited a significantly higher rate of distal recurrences than the external control cohort in multivariable analyses accounting for differences in clinical factors between the cohorts, acknowledging some differences in the methodology of determining pattern of failure based on the available information for each cohort (Supplementary Figure 3D–E). Importantly, these factors include bevacizumab therapy, which can increase the rate of distal progression in addition to confounding target volume delineation, potentially increasing the risk of marginal recurrences when treating enhancing disease.^27^ Taken together, these analyses suggest that our novel schedule may achieve improved tumor control (Appendix 5). However, this very small trial was not powered to measure efficacy. An appropriately powered randomized trial, beyond the scope of this study, is necessary to make any definitive conclusions regarding efficacy.

Our study possesses several limitations. First, the treatment-naïve mouse model used in our preclinical study may fail to capture the relevant biology of the heavily pretreated population of patients included in our trial. Second, while we could have benchmarked our treatment regimen against more dose-intensive or stereotactic treatment schedules, we aimed to conduct this first-in-human study as referenced to a well-established regimen (35 Gy in 10 fractions) with no prior reported grade 4/5 toxicity as observed within a prospective, randomized multi-institutional study.^22^ Third, we did not perform any clinical validation of the effect of our novel schedule on cell-state plasticity in our trial, because it is rarely possible to collect tumor tissue shortly following re-irradiation of recurrent glioblastoma patients. Such a study could be performed in the future using a window of opportunity trial design in patients due to undergo surgical resection.^28^ Fourth, this study evaluated a small (*n* = 14) cohort with significant heterogeneity in prior treatment and baseline prognostic factors without a control arm for robust assessment of the efficacy of our schedule, which was beyond the scope of our study aiming to first assess the feasibility and safety of our nonstandard schedule before embarking on a larger study. We plan on performing such a randomized controlled trial in the future with metabolic and imaging response assessment, pending further mechanistic preclinical investigation to identify the most suitable patients and treatment response biomarkers and determine whether patients could benefit from personalized administration schedules. Based on our previous findings that an alternative scheduling strategy is superior in the context of concurrent temozolomide,^20^ we intend to focus future study of model-adapted radiation towards cohorts of patients receiving re-irradiation without concurrent temozolomide, with progression-free survival and overall survival as the primary endpoints. In theory, future studies may also explore model-adapted radiation monotherapy for newly diagnosed patients with unmethylated MGMT promoters or for older and infirm patients. Finally, our feasibility trial was conducted at a single center, and it is possible that our feasibility result will not generalize to other centers and clinical scenarios.

In conclusion, we here provide evidence suggesting that a novel radiation therapy schedule, exploiting cell-state plasticity, holds promising clinical potential. We have demonstrated that this novel therapeutic approach is feasible and safe to administer to recurrent GBM patients. We believe that the promise, feasibility, and low cost of our therapeutic strategy warrant further mechanistic investigations using preclinical models to guide a future efficacy trial in patients.

## Supplementary Material

noac253_suppl_Supplementary_Material
