# Supplementary material for: Phase I study of a novel glioblastoma radiation therapy schedule exploiting cell-state plasticity
Source: Neuro Oncol. 2022 Nov 19;25(6):1100–12. doi: 10.1093/neuonc/noac253 (PMC10237407; doi:10.1093/neuonc/noac253)
Supplement: noac253_suppl_Supplementary_Material [file noac253_suppl_supplementary_material.docx]

**Supplemental material**

**Appendix 1: A novel radiation schedule is predicted to provide a clinically meaningful survival benefit**

We first sought to understand how the mouse model used in our preclinical research^1^ represents treatment outcomes of GBM patient populations. We developed two linear regression models to estimate the survival improvement for patients resulting from the optimized radiation therapy schedule. These statistical models are based on the relationship between survival improvement, expressed as hazard ratio, achieved by different interventions in preclinical studies (employing the same mouse model as our preclinical study^1^) and survival of patients in clinical trials testing the same interventions (Supplementary Fig. 1A).

We obtained data to fit the models by performing a literature search to identify all published studies using the same mouse model as used in our preclinical study to compare different treatment approaches that have also been tested in randomized trials in GBM patients. For treatment comparisons for which no hazard ratios were provided in the publication, we extracted the survival data from the Kaplan Meier curves using WebPlotDigitizer. Cox proportional hazards regression models were fit to these data using Firth’s penalized likelihood and profile penalized likelihood confidence intervals, as implemented in the coxphf R package. We excluded studies for which insufficient data were available to calculate the hazard ratio. The interventions assessed in both mice and patients include the use of radiation therapy with standard fractionation, hyperfractionation, hypofraction, dose escalation, temozolomide-radiation combination therapy and bevacizumab-radiation combination therapy (Supplementary Table 1).

Using these data, we fit a linear regression model of the natural logarithm of the hazard ratio for overall survival in the clinical trial against the natural logarithm of the hazard ratio for overall survival in the matched preclinical trials (Model 1). The model for human overall survival hazard ratio prediction is given by the expression

$$\begin{aligned} {ln(HR}_{human})={\beta_{0}+\beta_{1}ln(HR}_{mouse})\#(1) \end{aligned}$$

where ${HR}_{human}$ and ${HR}_{mouse}$ are the hazard ratios for overall survival for GBM patients and the mouse model, respectively, and $\beta_{0}$ and $\beta_{1}$ are the regression coefficients. The model was fit to the data using the stats R package.

Estimated effect sizes from preclinical studies are often inflated due to small sample sizes, leading to the so-called ‘winner’s curse’ phenomenon^2^. The mouse hazard ratio estimates in isolation do not capture the sample size or inter-mouse variability for the corresponding experiments. The upper 95% confidence interval of the mouse hazard ratio gives a more conservative estimate of the true mouse hazard ratio, accounting for sample size and variability between mice. Therefore, we generated a second model using the upper 95% confidence interval of the mouse trial hazard ratio to predict the hazard ratio for the corresponding clinical trial (Model 2). As there is considerable uncertainty in performing model selection, especially for small numbers of data points, we employed a model averaging approach when considering the clinical potential of novel interventions. Specifically, we investigated the mean of the predictions of both models, with equal weighting between models. We used this approach in preference to generating a single model with the mouse hazard ratio and the upper 95% confidence interval as covariates as these two values share a dependence, which is an unfavorable property for linear regression modeling. The models and data used to construct them are available at <https://github.com/jamiedean/glioblastoma-radiation-therapy-schedule>.

Of the 7 different interventions tested in the mouse model and randomized clinical trials, 3 showed a statistically significant survival benefit in humans. Note that the fact that the confidence intervals for the hazard ratios for the other 4 interventions include 1 does not imply that those hazard ratios are equivalent. Also, some of the mouse experiments corresponding to those “negative” trials showed significant survival differences. Therefore, hazard ratios for these interventions still provide effect size measures that inform the quantification of the correlation between mouse and human treatment responses. The first regression model is based on the measured hazard ratio of treatment comparisons in the mouse model (Model 1). The second is based on the upper 95% confidence interval of the hazard ratio from the mouse trials (Model 2), which we developed as a more conservative estimate of the effect size after noting that the smaller mouse trial hazard ratio estimates had wider confidence intervals (Supplementary Fig. 1A). Both of these metrics correlated highly with the human trial hazard ratios (Pearson correlation coefficients = 0.91 and 0.88, p-values = 0.0002 and 0.0007, respectively; correlations performed on the logarithms of the hazard ratios). To provide perspective, this correspondence is substantially higher than previously measured correlations between xenograft and phase II trial treatment responses, which were relatively poor^3^. We found that our models provided an accurate fit to the data (Model 1, Adjusted R^2^ = 0.80; Model 2, Adjusted R^2^ = 0.75) and indicated statistically significant associations between the mouse and human hazard ratios (Model 1, p = 0.0003; Model 2, p = 0.0008; Supplementary Tables 2 and 3). Using this approach, we found that the hazard ratios in the mouse studies consistently overestimated the magnitude of benefit observed in clinical trials. This observation could be due to all patients benefiting less from the experimental interventions than in the mouse model, some patients deriving the same benefit observed in the mouse model (or more) and others no benefit, or a combination of both. Our statistical models, quantifying this bias, enable us to predict the human hazard ratios of interventions that have been evaluated in mouse models.

We used our statistical models to predict the human hazard ratio for death for the optimized versus standard radiation therapy schedule to be 0.74 (Supplementary Fig. 1B, Supplementary Table 4). For comparison, we also analyzed GBM clinical trials evaluating novel therapies that are in progress and have been tested in the same mouse model. We identified clinical trials evaluating the PARP inhibitor veliparib (NCT02152982, NCT00946335, NCT03581292, NCT00770471, NCT01514201), the anti-PD-1 immune checkpoint inhibitor pembrolizumab (NCT03661723) and the anti-PD-L1 immune checkpoint inhibitors durvalumab (NCT02866747, NCT02336165) and avelumab (NCT02968940) as meeting these criteria. Veliparib^4^, anti-PD-1, and anti-PD-L1^5^ have been tested in the same mouse model that we used in our preclinical study and that was used to develop our statistical model to predict human trial hazard ratios from mouse trial hazard ratios. Our novel radiation therapy schedule is predicted to provide a greater survival benefit than the addition of veliparib, anti-PD-1, or anti-PD-L1 to current standard-of-care therapies (Supplementary Fig. 1B, Supplementary Table 4). While the relative ranking of these interventions can be predicted by comparing the mouse trial hazard ratios without the need for a model, our statistical models provide additional useful information to inform which interventions to progress to clinical testing. Specifically, our models provide estimates of the human trial hazard ratios, which can then be compared to clinically meaningful effect sizes to determine whether an intervention is promising enough to evaluate in patients. The magnitude of the predicted benefit of our novel radiation schedule is similar to the magnitude of benefit that phase III trials of GBM interventions are commonly powered to detect. For example, the EORTC 26981/22981 trial evaluating the benefit of the addition of temozolomide to standard-of-care therapy was powered to detect a hazard ratio for death of 0.75^6^. We therefore concluded that our novel intervention possessed sufficient promise to warrant clinical evaluation.

Following the completion of our clinical trial, the results of the Alliance A071102 trial, evaluating veliparib and temzolomide versus placebo and temozolomide, were reported in abstract form^7^. This provided an opportunity to prospectively evaluate the predictive performance of our statistical models. Our models predict an overall survival hazard ratio for veliparib and temozolomide versus temozolomide of 0.83 (mean of Model 1 and Model 2 predictions; Model 1 prediction = 0.86, Model 2 prediction = 0.81) (Supplementary Table 4). These are the hazard ratios that any trial would have to be powered for to detect a statistically significant overall survival benefit. The Alliance A071102 trial was powered to detect an overall survival hazard ratio of 0.71^7^. Our statistical models therefore correctly predicted that this trial would not observe a statistically significant overall survival benefit. The trial reported an overall survival hazard ratio of 0.89 (95% confidence interval = 0.71 – 1.11), close to that predicted by our statistical models. Extending our mathematical model of glioblastoma cell population dynamics^1,8^ to incorporate the effects of PARP inhibitors in order to study their optimal use is the focus of ongoing work.

**Appendix 2: The time interval between radiation administrations required to delay tumor progression appears consistent across species**

Our preclinical study revealed that an approximately 3.25 ± 1.5 hour time interval between radiation fractions was necessary to achieve the survival advantage of optimized versus standard schedules^1^. The proposed mechanism responsible for the survival advantage is an increase in the rate of dedifferentiation of rapidly proliferating differentiated cells to less proliferative stem-like glioma cells when such an interfraction interval is used. However, it is not known whether the association between this interfraction interval and increased survival is a general phenomenon or specific to the particular mouse model used. Therefore, before translating the novel schedule to patients, we assessed whether this association was robust across different experimental systems and species.

We performed a literature search to identify all published preclinical and clinical studies directly comparing the effect of the time interval between radiation administrations on survival or tumor volume dynamics in malignant glioma. We excluded studies where the majority of the course of radiation was administered in combination with any chemotherapy as concurrent drug and radiation administration would not result in 3 – 4 h intervals between cells experiencing the induction of new DNA damage. We also excluded single arm studies comparing to historical controls due to the high risk of bias. We tested the association between interfraction intervals predicted to improve survival and measured tumor control or survival using Fisher’s exact test, due to the small number of studies. Code and data for our analyses are available at https://github.com/jamiedean/glioblastoma-radiation-therapy-schedule.

We identified eight *in vivo* preclinical comparisons, employing human cell line heterotopic xenografts, a genetically engineered mouse model or a rat model^1,9–12^, and eight clinical comparisons^13–20^ of the effect of different interfraction intervals on malignant glioma radiation response (Supplementary Fig. 4A). This data focuses on de novo GBM since no similar data is available, to our knowledge, for recurrent GBM. Thirteen of 16 comparisons (81%) used an interfraction interval predicted to lead to a survival advantage (3 – 4.75 hours). Of these, 5/7 (71%) preclinical comparisons^1,9,10^ and 4/6 (67%) clinical comparisons (two of which are randomized comparisons)^14,15,17,19^ indeed showed an improved tumor response with a 3 – 4.75 hour interfraction interval and the remaining 2/6 clinical comparisons exhibited a trend towards significance^13,20^. The two preclinical comparisons failing to show a survival advantage despite predictions to the contrary used a dose per fraction (0.4 Gy) of less than half of that used in our preclinical study (1.0 Gy)^1^. The preclinical studies that did demonstrate the predicted survival improvement used doses per fraction (0.7 Gy and 0.8 Gy) closer to those used in our preclinical study (1.0 Gy). One of the preclinical studies and two of the clinical studies used longer interfraction intervals [6 – 8 hours^11,16,18^] than those predicted to provide a benefit. In 3/3 (100%) of these studies there was no statistically significant association between interfraction interval and tumor control.

Combining these observations indicates that 9/13 of comparisons (69%) predicted to show superiority did and 3/3 of comparisons (100%) predicted not to show superiority did not, representing a trend towards statistical significance (p = 0.06, Fisher’s exact test) in this small sample. Our analysis thus suggests that a 3 – 4.75 hour interfraction interval may delay time to tumor progression compared with an interval of 5.75 hours or higher in mice, rats, and humans. This finding serves to increase the evidence that utilizing a 3.25 hour interval between radiation administrations is a promising strategy that warrants clinical investigation.

A limitation of our analysis of the association between interfraction interval and survival is the assumption that a 3.25 hour interfraction interval is always predicted to meaningfully improve survival, irrespective of the other details of the administration schedule. However, this is not the case as demonstrated by our mathematical modeling (Fig. 3A). Therefore, previous studies employing approximately 3.25 hour interfraction intervals (Supplementary Fig. 4A) may result in smaller benefits than those that could be achieved by a schedule based on a quantitative understanding of dynamic heterogeneity and plasticity of GBM radiation response. We did not use our mathematical model to directly predict the outcomes of the previous schedules tested due to insufficient data being available to reparametrize the model for different experimental systems and species.

A more direct preclinical experimental investigation into the relationship between the time interval between radiation fractions and treatment efficacy will be performed in the future, subject to the successful demonstration of feasibility and safety of administering a non-standard radiation therapy schedule. This will explore the molecular mechanism underlying the effect and whether it varies between species and between patients. This will inform the design of future efficacy trials.

**Appendix 3: Enriching tumors for stem-like cells is not associated with decreased survival**

Our preclinical study suggests that enriching tumors for radioresistant, yet slowly proliferating, stem-like cells increases survival^1^. Employing a treatment strategy that deliberately enriches tumors for treatment resistant cell populations could potentially worsen the survival of patients. Therefore, before treating any patients using our novel schedule, we sought to use existing data to measure the association of stem-like cell dynamics and treatment response in patients. Specifically, we sought to determine the relationship between enrichment for glioma stem-like cells and survival in patients. We performed a literature search to find studies that measured the glioma stem-like cell fraction before and after radiation therapy as well as the time to tumor progression or death. We identified two cohorts meeting these criteria, one from Italy and one from Japan^21–23^. Both identified glioma stem-like cells by the commonly used CD133 marker, which selects for cells with stem cell-like and tumor-initiating properties^24,25^, was employed to identify glioma stem cells.

We extracted data from these studies and obtained previously unpublished data from the study authors where necessary (Japanese cohort). We fit multivariable Cox proportional hazards regression models to the data to test the associations between the stem-like cell fraction at either initial surgery or recurrence surgery or the change in the stem-like cell fraction between the two surgeries and recurrence-free survival, survival following recurrence and overall survival using the coxphf R package. We also included all available prognostic factors as covariates, including age, sex, Karnofsky Performance Status, radiation dose, use of temozolomide, use of any chemotherapy and MGMT methylation status.

When analyzing the effect of the change of a measurement on an outcome of interest it is important to minimize the dependence of the change in the measurement on its baseline value. To assess this dependence, we visualized the change in stem-like cell fraction using Bland-Altman plots. Where there was a dependence of the change in stem-like cell fraction on the initial value we attempted to minimize this dependence through transforming the CD133+ fraction. We tried different transformations and selected those which resulted in the smallest dependence (Italian cohort: natural logarithm; Japanese cohort: fifth root). Code and data for our analyses are available at https://github.com/jamiedean/glioblastoma-radiation-therapy-schedule.

The first dataset comprised an Italian cohort of 37 GBM patients uniformly treated with surgery, radiation and temozolomide^23^. The stem-like cell number was inferred by measuring the CD133+ cell fraction in the primary surgical specimen and the specimen from a second surgical resection at recurrence. We performed multivariable Cox proportional hazards regression analyses including the change in CD133+ cell fraction and available prognostic factors (age, sex, MGMT methylation status and Karnofsky Performance Status) as covariates and found a highly statistically significant association between the change in CD133+ cell fraction and increased overall survival (HR = 0.42; p = 0.00029) (Supplementary Fig. 4B). The change in CD133+ cell fraction was also highly significantly associated with survival following recurrence surgery (HR = 0.49; p = 0.00085) (Supplementary Fig. 4C) and exhibited a trend towards a significant association with recurrence-free survival (HR = 0.67; p = 0.074) (Supplementary Fig. 4D). Neither the CD133+ cell fraction before nor after treatment was significantly associated with survival (Supplementary Fig. 5A - F). This observation suggests that the change in the stem-like cell fraction, rather than the absolute fraction pre- or post-treatment, correlates with survival.

The second dataset comprised a Japanese cohort of 20 GBM patients treated with surgery, radiation and radiosurgery^21,22^. The use of chemotherapy and the radiation dose administered were heterogeneous. The CD133+ cell fraction was measured in the primary surgical specimen and the specimen from the surgical resection at recurrence. We performed multivariable Cox proportional hazards regression analyses including available prognostic factors (age, sex, chemotherapy administration and radiation dose) as covariates and found a significant association between the fraction of CD133+ cells following radiation therapy and recurrence-free survival (HR = 0.92; p = 0.011) (Supplementary Fig. 4E). The association remained significant upon replacing chemotherapy administration with temozolomide administration as a covariate (HR = 0.94; p = 0.037) (Supplementary Fig. 5G). In contrast to the first dataset, there were no significant associations between the increase in CD133+ cell fraction (HR = 2.0; p = 0.31) (Supplementary Fig. 5H) or the CD133+ cell fraction at primary surgery and recurrence-free survival (HR = 0.81; p = 0.17) (Supplementary Fig. 5I). Overall survival data were not available for analysis.

These analyses are in agreement with the proposed mechanism for the improved survival in our preclinical study^1^ – employing a treatment strategy that increases the fraction of stem-like cells increases survival. However, we stress that these findings are not intended as robust clinical evidence supporting the proposed mechanism due to several important limitations (discussed below). Rather these analyses were intended to test whether employing a treatment strategy that deliberately attempts to increase the fraction of stem-like cells will be harmful to patients, using existing data, before treating any patients using our novel schedule. Importantly, in neither cohort were we able to find any statistically significant associations between a higher fraction of CD133+ cells or increase in CD133+ cell fraction and worse clinical outcomes (with any combination of covariates; data not shown). This increased our confidence that intentionally enriching tumors for stem-like cells is unlikely to decrease survival in GBM patients. We, therefore, decided that our schedule was appropriate for feasibility and safety testing in a small cohort of patients.

Regarding the interpretations of the results as they pertain to the proposed mechanism underlying the preclinical efficacy of the schedule, it should be noted that for one cohort the increase in the CD133+ cell fraction is associated with clinical outcomes, while for the other cohort it is the CD133+ cell fraction at recurrence. Moreover, the correlation between CD133+ cell fraction and treatment response could alternatively be explained by superior killing of CD133- cells. These analyses possess substantial weaknesses that limit their interpretation to no more than hypothesis-generating. First, the size of both cohorts is very small, limiting the robustness of the results. Second, the data are subject to sampling bias and the samples obtained may not be representative of the whole tumor. Third, the datasets of patient glioma stem-like cell dynamics we analyzed (Supplementary Fig. 4B - E) used CD133 as marker of glioma stem cell identity. However, staining tumor tissue for CD133 does not provide a functional measure of stemness. Also, although CD133 is highly relevant to stemness^24^, radiation response^26^ and patient prognosis^27^, it is not a universal glioma stem-like cell marker. Since these datasets were generated it has been shown that CD133 is a marker of proneural glioma stem-like cells, but not mesenchymal glioma stem-like cells^28^. Therefore, the association between increased survival and increase in CD133+ cell fraction or CD133+ fraction post-treatment could be indicative of the extent of proneural-mesenchymal transition, which is associated with survival^28^. Additional, markers of non-overlapping populations of glioma stem-like cells have been identified, including CD44 and CD49f, which we could not analyze due to lack of available data. These preliminary hypothesis-generating results will help to inform the design of a future clinical study, utilizing a window of opportunity design to investigate the effect of the schedule on stem-like cell dynamics in patients, which will incorporate multiple different glioma stem-like cell markers.

**Appendix 4: Improving the selection of preclinical discoveries to translate into clinical trials**

Given that the majority of promising preclinical findings fail to translate into improved patient outcomes^29^, improvements are needed in the decision-making process for choosing whether to advance a preclinical finding into clinical evaluation. The aim of our approach is to filter out non-promising interventions through a rigorous quantitative investigation of clinical potential^30^. To this end, we developed the first predictive model of GBM clinical trial outcomes (Supplementary Fig. 1). This model highlights that mouse trials systematically overestimate the benefit of interventions evaluated in clinical trials. We quantified this bias to provide quantitative predictions of clinical trial outcomes. The model serves as a useful tool to the neuro-oncology community and will be built upon as more mouse and human trial data become available. We believe that broad application of our methods may reduce the high failure rate of clinical trials and focus resources towards the most promising interventions. Furthermore, this approach may work complementarily with efforts to incorporate real-world clinical trial designs focused on more diverse patient populations and clinical settings.

**Appendix 5: Additional discussion of the potential difference in timing and pattern of progression**

Our analyses indicated improved progression-free survival and a lower rate of local recurrence in patients treated on our trial compared with an external control arm. The inferred magnitude of these effects is large, allowing it to be detected even in our small cohort. We attempted to minimize potential confounding, such as the use of bevacizumab therapy, by performing a multivariable statistical comparison. However, the small sample size and potential for residual confounding should be considered in the interpretation of these observations.

While this potential advantage did not translate into an overall survival benefit in our small sample, the findings may indicate that our novel schedule provides superior tumor control than conventionally used re-irradiation schedules. It remains to be seen whether an improved overall survival would be achieved in an appropriately powered efficacy trial. However, alternative explanantions for the apparent improvement in local control are also consistent with our data. Considering our preliminary findings, it is important to note that in our preclinical study mice were treated with whole brain radiation, whereas patients receive involved field radiation only. This difference provides two additional potential explanations for why we observed a substantial survival advantage in the preclinical model^1^, but no obvious overall survival benefit in our small single-arm trial. One explanation is that the optimized schedule may improve control of tumor cells within the radiation treatment volume, but that local control does not influence overall survival in recurrent GBM patients. In support of this explanation, early results from a randomized trial evaluating the benefit of adding radiation to bevacizumab for recurrent GBM patients found that radiation improved 6 month progression-free survival, but not overall survival^31^. A second explanation is predicated on the basis that glioma stem-like cells exhibit reduced proliferation in response to radiation^32^ and exhibit increased invasion compared with differentiated cells^33^. Our novel schedule may steer cells into a stem-like state, facilitating their migration, with reduced proliferation, out of the radiation treatment volume in patients, after which time they start to rapidly proliferate again. In mice receiving whole brain radiation the cells never leave the high dose region and so do not start rapidly proliferating again. This explanation is consistent with previous studies demonstrating that distal recurrences are associated with expression of the stem cell marker CD133^34–36^.

Reverse-translation of these preliminary findings is merited to robustly test these hypotheses in preclinical models. Such a study additionally has the potential to inform a novel target volume definition. In particular, the high rates of marginal disease failure may indicate potential opportunities to optimize target delineation beyond strictly enhancing disease, in the post-bevacizumab treatment setting, to attempt to convert this potential impact on tumor biology into an increase in survival for patients. For example, inclusion of the FLAIR signal within re-treatment volumes may prove particularly viable with fractionated approaches for re-irradiation such as this regimen.

**Supplemental figures**

**Supplementary Fig. 1.** Statistical modeling of the clinical efficacy of GBM therapies suggests that our novel radiation schedule has promising clinical potential. **(A)** Linear regression models of the measured human trial overall survival hazard ratio against the measured mouse trial survival hazard ratio (Model 1) and the upper 95% confidence interval of the preclinical trial survival hazard ratio (Model 2). **(B)** Predictions (mean of Model 1 and Model 2 predictions) for the human hazard ratios of novel interventions currently under evaluation in clinical trials based on their measured mouse trial hazard ratios. The color bar shows the upper 95% confidence intervals measured in the mouse trials. Model HR – hazard ratio; CI – confidence interval; RT – radiation therapy; TMZ – temozolomide; PARPi - veliparib.

**Supplementary Fig. 2.** Mathematical model predicted sensitivity of tumor response to alterations from the prescribed administration schedule. **(A)** Illustration of the proposed cell fate dynamics in response to the novel administration schedule. The first phase of the schedule reduces the tumor population size through predominantly killing radiosensitive cells and the second phase reprograms the remaining cells towards a slowly proliferating stem-like state, increasing the time to progression and death. **(B)** Predicted effect of different interfraction intervals on tumor volume dynamics for three times daily administrations. **(C)** Predicted effect of different interfraction intervals on tumor volume dynamics for once daily administrations. **(D)** Predicted effects of starting the schedule on different days of the week. For all panels, all curves apart from the one corresponding to the 3.5 Gy x 10 (QD) schedule correspond to the optimized schedule (3.96 Gy x 7 (QD) + 1.0 Gy x 9 (TID)) with different interfraction intervals or starting days. QD – once daily dosing; TID – three times daily dosing.

**Supplementary Fig. 3.** Effect of the novel radiation schedule on overall survival and recurrence location. **(A)** Comparison of the distributions of clinical covariates in the phase I trial cohort (Experimental) and external control cohort. **(B)** Ratio of time to progression following re-irradiation and prior line of therapy. **(C)** Ratio of time to progression following re-irradiation and first line therapy coding marginal failures in the trial as local failures. **(D)** Logistic regression odds ratios for distant failure. **(E)** Logistic regression odds ratios for distant failure coding marginal failures in the trial as distant. RT – radiation therapy; BED – biologically effective dose; KPS – Karnofsky performance status; Re-RT – reirradiation; TTP – time to progression.

**Supplementary Fig. 4.** A specific interfraction interval and enrichment of CD133+ cells following radiotherapy are associated with improved outcomes. **(A)** Comparison of the effect of different interfraction intervals on survival in model systems and patients. The size of the points represents the relative superiority (larger points), inferiority (smaller points) or equivalence (same size points) of the compared schedules. The pink shaded region shows the range of interfraction intervals predicted to provide a survival advantage. **(B - D)** Kaplan-Meier plots showing the association between the increase in CD133+ cell fraction following radiation and temozolomide therapy and **(B)** overall survival **(C)** time from recurrence surgery to death and **(D)** time to recurrence in an Italian GBM patient cohort ^23^. The cohort is dichotomized at the median increase in CD133+ cell fraction value for visualization. The reported p-value is from a Cox proportional hazards regression model of survival against CD133+ change represented as a continuous variable. **(E)** Forest plot showing the association between the CD133+ cell fraction following radiation therapy and time to recurrence surgery in a Japanese GBM patient cohort ^21,22^. KPS – Karnofsky Performance Status.

**Supplementary Fig. 5.** Association between stem-like cell dynamics and survival in GBM patient cohorts. **(A - C)** Multivariable Cox proportional hazards regression model of **(A)** overall survival, **(B)** time from recurrence to death and **(C)** time to recurrence with the CD133+ cell fraction at primary surgery in an Italian GBM patient cohort ^23^. **(D - F)** Multivariable Cox proportional hazards regression model of **(D)** overall survival, **(E)** time from recurrence to death and **(F)** time to recurrence with the the CD133+ cell fraction at recurrence surgery in an Italian GBM patient cohort ^23^. (**G - I)** Multivariable Cox proportional hazards regression model of time to recurrence with **(G)** temozolomide, **(H)** the change in the CD133+ cell fraction or **(I)** the CD133+ cell fraction at primary surgery as covariates in a Japanese GBM patient cohort ^21,22^. KPS – Karnofsky Performance Status.

**Supplemental tables**

**Supplementary Table 1.** Matched mouse and human trial survival data used to contruct the statistical models to predict human survival from mouse survival data.

| Intervention | Mouse trial overall survival hazard ratio (95% CI) | Mouse trial reference | Human trial overall survival hazard ratio (95% CI) | Human trial reference |
| --- | --- | --- | --- | --- |
| Radiation therapy versus Untreated | 0.016 (0.00012 – 0.13) | Leder *et al.* 2014^1^ | 0.47 (0.29 – 0.76) | Keime-Guibert *et al.* 2007^37^ |
| Hypofractionated radiation therapy versus Standard fractionation radiation therapy | 0.34 (0.11 – 1.05) | Leder *et al.* 2014^1^ | 0.85 (0.64 – 1.12) | Malmstrom *et al.* 2012^38^ |
| Hypofractionated radiation therapy versus Standard fractionation radiation therapy | 0.34 (0.11 – 1.05) | Leder *et al.* 2014^1^ | 0.89 (0.59 – 1.34) | Roa *et al.* 2004^39^ |
| Hyperfractionated radiation therapy versus Standard fractionation radiation therapy | 0.52 (0.17 – 1.17) | Leder *et al.* 2014^1^ | 1.1 (0.86 – 1.4) | Prados *et al.* 2001^16^ |
| Dose escalated radiation therapy versus Standard radiation therapy | 0.047 (0.00035 – 0.45) | Lemasson *et al.* 2013^40^ | 0.81 (0.66– 0.99) | Bleehen *et al.* 1991^41^ |
| Temozolomide + Radiation therapy versus Radiation therapy | 0.061 (0.00046 – 0.51) | Lemasson *et al.* 2016^40^ | 0.6 (0.5 – 0.7) | Stupp *et al.* 2009^42^ |
| Temozolomide + Radiation therapy versus Radiation therapy | 0.061 (0.00046 – 0.51) | Lemasson *et al.* 2016^40^ | 0.67 (0.56– 0.80) | Perry *et al.* 2017^43^ |
| Temozolomide + Radiation therapy versus Radiation therapy | 0.061 (0.00046 – 0.51) | Lemasson *et al.* 2016^40^ | 0.66 (0.53 – 0.83) | Athanassiou *et al.* 2005^44^ |
| Radiation therapy versus Temozolomide | 2.77 (1.19 – 6.49) | Lemasson *et al.* 2016^40^ | 1.09 (0.84 – 1.42) | Wick *et al.* 2012^45^ |
| Bevacizumab + Radiation therapy versus Radiation therapy | 0.87 (0.33 – 2.48) | Pitter *et al.* 2016^46^ | 1.09 (0.63 – 1.89) | Wirsching *et al.* 2018^47^ |

**Supplementary Table 2.** Results of the model fitting for Model 1.

| Coefficient | Estimate | Standard error | p-value |
| --- | --- | --- | --- |
| Intercept, $\beta_{0}$ | 0.055 | 0.061 | 0.39 |
| Mouse trial hazard ratio, $\beta_{1}$ | 0.16 | 0.026 | 0.00027 |
| Adjusted R^2^ | 0.80 | | |

**Supplementary Table 3.** Results of the model fitting for Model 2.

| Coefficient | Estimate | Standard error | p-value |
| --- | --- | --- | --- |
| Intercept, $\beta_{0}$ | -0.19 | 0.046 | 0.0037 |
| Mouse trial hazard ratio, $\beta_{1}$ | 0.24 | 0.045 | 0.00074 |
| Adjusted R^2^ | 0.75 | | |

**Supplementary Table 4.** Model predictions of novel GBM interventions currently undergoing clinical evaluation.

| Intervention | Clinical trials | Mouse trial overall survival hazard ratio (95% CI) | Predicted human overall survival hazard ratio | | |
| --- | --- | --- | --- | --- | --- |
|  |  |  | Model 1 | Model 2 | Mean |
| Veliparib + Temozolomide versus Temozolomide | NCT02152982^7^, NCT00946335 | 0.28 (0.07 – 0.89)^4^ | 0.86 | 0.81 | 0.83 |
| Veliparib + Temozolomide + Radiation therapy versus Temozolomide + Radiation therapy | NCT03581292, NCT00770471, NCT01514201^48^, ACTRN12615000407594^49^ | 0.14 (0.01 – 0.79)^4^ | 0.77 | 0.78 | 0.78 |
| Pembrolizumab + Radiation therapy versus Radiation therapy | NCT03661723 | 1.23 (0.44 – 3.34)^5^ | 1.09 | 1.11 | 1.10 |
| Durvalumab or Avelumab + Radiation therapy versus Radiation therapy | NCT02866747, NCT02336165, NCT02968940 | 0.35 (0.12 – 0.96)^5^ | 0.89 | 0.82 | 0.86 |
| Optimized radiation therapy schedule versus Standard radiation therapy schedule | Proposed in the present study | 0.27 (0.04 – 0.30)^1^ | 0.85 | 0.62 | 0.74 |

**Supplementary Table 5.** Assessments completed at each visit (up to long term follow-up 3).

| **Assessment** | **Baseline** | **Treatment Week 1** | **Treatment Week 2** | **Four Week Follow-Up** | **Long Term Follow-Up 1** | **Long Term Follow-Up 2** | **Long Term Follow-Up 3** |
| --- | --- | --- | --- | --- | --- | --- | --- |
| Physical Exam | 14 | 13 | 11 | 10 | 7 | 7 | 6 |
| Neurological Exam | 14 | 14 | 13 | 10 | 9 | 9 | 8 |
| KPS | 14 | 14 | 13 | 10 | 8 | 8 | 7 |
| MDASI | 8 | 7 | 5 | 6 | 2 | 2 | 2 |
| Radiologic Imaging | 13 | 0 | 1 | 10 | 10 | 9 | 8 |

**Supplementary Table 6.** Tumor dose coverage objectives (given as absorbed dose) for phases 1 and 2 combined.

| Dose Metric | Per Protocol | Variation Acceptable | Deviation Unacceptable |
| --- | --- | --- | --- |
| Volume of PTV covered by the prescription dose 36.72 Gy | ≥ 95% of the PTV should receive the prescription dose or higher | ≥ 90% of the PTV should receive the prescription dose or higher | < 90% of the PTV should receive the prescription dose or higher |
| Minimum dose to the PTV (0.03 cc) | ≥ 85% of the prescription dose (31.21 Gy) | ≥ 80% of the prescription dose (29.38 Gy); minimum doses < 80% of the prescription dose are permissible if they occur at an area of overlap with an organ at risk (OAR) | < 80% of the prescription dose (29.38 Gy); minimum doses < 80% of the prescription dose are unacceptable if they do not occur at an area of overlap with an OAR |
| Maximum dose to the  PTV (0.03 cc) | ≤ 120% of the prescription dose (44.06 Gy) | ≤ 130% of the prescription dose (47.74 Gy) | > 130% of the prescription dose (47.74 Gy) |

PTV – Planning target volume.

**Supplementary Table 7.** Normal tissue dose constraints (given as biologically effective dose with an alpha/beta ratio of 2) for phases 1 and 2 combined.

| Dose Metric | Per Protocol | Variation Acceptable | Deviation Unacceptable |
| --- | --- | --- | --- |
| Scenario (1): Previous radiation to the local area including critical organs at risk | | | |
| Maximum Dose to PRV for Optic Nerves and Chiasm (D_0.03 cc_) | Less than or equal to 40.00 Gy_2_ | Greater than 40.00 Gy_2_ but less than or equal to 56.25 Gy_2_ | Greater than 56.25 Gy_2_ |
| Maximum Dose to Brainstem (0.03 cc) | Less than or equal to 52.80 Gy_2_ | Greater than 52.80 Gy_2_ but less than or equal to 75.00 Gy_2_ | Greater than 75.00 Gy_2_ |
| Scenario (2): No previous radiation to the local area or critical organs at risk | | | |
| Maximum Dose to PRV for Optic Nerves and Chiasm (0.03 cc) | Less than or equal to 96.11 Gy_2_ (the prescription dose) | Greater than 96.11 Gy_2_ but less than or equal to 104.28 Gy_2_ | Greater than 104.28 Gy_2_ |
| Maximum Dose to Brainstem (0.03 cc) | Less than or equal to 96.11 Gy_2_ (the prescription dose) | Greater than 96.11 Gy_2_ but less than or equal to 104.28 Gy_2_ | Greater than 104.28 Gy_2_ |

PRV – Planning organ-at-risk volume.

**Supplementary Table 8.** Characteristics of patients in the external control cohort.

| **Variable** | **N = 92** |
| --- | --- |
| **Age (years)** |  |
| Median (range) | 59.6 (30.0 - 93.0) |
| Mean (sd) | 58.4 (10.7) |
| **Sex** |  |
| Female | 37 (40.2%) |
| Male | 55 (59.8%) |
| **KPS** |  |
| 100 | 2 (2.2%) |
| 90 | 31 (33.7%) |
| 80 | 33 (35.9%) |
| 70 | 14 (15.2%) |
| 60 | 3 (3.3%) |
| Unknown | 9 (9.8%) |
| **Time from initial diagnosis to re-irradiation (months)** |  |
| Median (range) | 18.0 (5.1 - 121.5) |
| Mean (sd) | 24.6 (19.8) |
| **Number of relapses (including present)** |  |
| 1 | 40 (43.5%) |
| 2 | 24 (26.1%) |
| 3 | 21 (22.8%) |
| 4 | 6 (6.5%) |
| 5 | 1 (1.1%) |
| **History of prior treatment with bevacizumab** |  |
| No | 59 (64.1%) |
| Yes | 33 (35.9%) |
| **Concurrent bevacizumab** |  |
| No | 49 (53.3%%) |
| Yes | 43 (46.7%) |
| **Tumor size* (mm^2^)** |  |
| Median (range) | 701.5 (0.0 - 5432.2) |
| Mean (sd) | 988.4 (1027.6) |
| **Radiation therapy biologically effective dose (Gy)** |  |
| Median (range) | 47.3 (23.6 - 78.0) |
| Mean (sd) | 49.6 (8.1) |

^*^Product of two perpendicular dimensions measured in millimeters. KPS – Karnofsky performance status.

**References**

1. Leder K, Pitter K, Laplant Q, et al. Mathematical modeling of PDGF-driven glioblastoma reveals optimized radiation dosing schedules. *Cell*. 2014;156(3):603-616. doi:10.1016/j.cell.2013.12.029

2. Button KS, Ioannidis JPA, Mokrysz C, et al. Power failure: Why small sample size undermines the reliability of neuroscience. *Nature Reviews Neuroscience*. 2013;14(5):365-376. doi:10.1038/nrn3475

3. Johnson JI, Decker S, Zaharevitz D, et al. Relationships between drug activity in NCI preclinical in vitro and in vivo models and early clinical trials. *British Journal of Cancer*. 2001;84(10):1424-1431. doi:10.1054/bjoc.2001.1796

4. Lemasson B, Wang H, Galbán S, et al. Evaluation of Concurrent Radiation, Temozolomide and ABT-888 Treatment Followed by Maintenance Therapy with Temozolomide and ABT-888 in a Genetically Engineered Glioblastoma Mouse Model. *Neoplasia*. 2016;18(2):82-89. doi:10.1016/j.neo.2015.11.014

5. Ene CI, Kreuser SA, Jung M, et al. Anti–PD-L1 antibody direct activation of macrophages contributes to a radiation-induced abscopal response in glioblastoma. *Neuro-Oncology*. 2019;22(December 2019):639-651. doi:10.1093/neuonc/noz226

6. Stupp R, Mason WP, van den Bent MJ, et al. Radiotherapy plus concomitant and adjuvant temozolomide for glioblastoma. *The New England journal of medicine*. 2005;352(10):987-996. doi:10.1056/NEJMoa043330

7. Sarkaria JN, Ballman K V., Kizilbash SH, et al. Randomized phase II/III trial of veliparib or placebo in combination with adjuvant temozolomide in newly diagnosed glioblastoma (GBM) patients with MGMT promoter hypermethylation (Alliance A071102). *Journal of Clinical Oncology*. 2022;40(16_suppl):2001-2001. doi:10.1200/jco.2022.40.16_suppl.2001

8. Randles A, Wirsching H georg, Dean JA, et al. Computational modelling of perivascular-niche dynamics for the optimization of treatment schedules for glioblastoma. *Nature Biomedical Engineering*. 2021;5(April). doi:10.1038/s41551-021-00710-3

9. Beauchesne PD, Bertrand S, Branche R, et al. Human malignant glioma cell lines are sensitive to low radiation doses. *International Journal of Cancer*. 2003;105(1):33-40. doi:10.1002/ijc.11033

10. Beauchesne P, Pedeux R, Bonmartin A, et al. Intracerebral glioma model in female hairless rats: assessment by using MRI and follow-up of irradiation. *Anticancer research*. 2003;23:3755-3760.

11. Krause M, Hessel F, Wohlfarth J, et al. Ultrafractionation in A7 human malignant glioma in nude mice. *Int J Radiat Biol*. 2003;79(6):377-383. doi:EABHEB7NGP15VU2B [pii]

12. Krause M, Wohlfarth J, Georgi B, et al. Low-dose hyperradiosensitivity of human glioblastoma cell lines in vitro does not translate into improved outcome of ultrafractionated radiotherapy in vivo. *International journal of radiation biology*. 2005;81(10):751-758. doi:10.1080/09553000500491537

13. Ludgate CM, Douglas BG, Dixon PF, Steinbok P, Jackson SM, Goodman GB. Superfractionated radiotherapy in grade III, IV intracranial gliomas. *International Journal of Radiation Oncology Biology Physics*. 1988;15:1091-1095.

14. Fulton DS, Urtasun RC, Shin KH, et al. Misonidazole combined with hyperfractionation in the management of malignant glioma. *Internation Journal of Radiation Oncology Biology Physics*. 1984;10(April):4-7.

15. Nelson DF, Curran WJ, Scott C, et al. Hyperfractionated radiation therapy and bis-chlorethyl nitrosourea in the treatment of malignant glioma - possible advantage observed at 72.0 Gy in 1.2 Gy B.I.D. fractions: report of the Radiation Therapy Oncology Group protocol 8302. *International Journal of Radiation Oncology, Biology, Physics*. 1993;25:193-207.

16. Prados MD, Wara WM, Sneed PK, et al. Phase III trial of accelerated hyperfractionation with or without difluromethylornithine (DFMO) versus standard fractionated radiotherapy with or without DFMO for newly diagnosed patients with glioblastoma multiforme. *International Journal of Radiation Oncology Biology Physics*. 2001;49(1):71-77. doi:10.1016/S0360-3016(00)01458-9

17. Jeremic B, Grujicic D, Antunovic V, Stojanovic M, Shibamoto Y. Hyperfractionated radiation therapy (HFX RT) followed by multiagent chemotherapy (CHT) in patients with malignant glioma: a phase II study. *International Journal of Radiation Oncology Biology Physics*. 1994;30(5):1179-1185.

18. Simpson WJ, Platts ME. Fractionation study in the treatment of glioblastoma multiforme. *International Journal of Radiation Oncology, Biology, Physics*. 1976;1(7-8):639-644. doi:10.1016/0360-3016(76)90145-0

19. Shin KH. Multiple daily fractionated radiation therapy and misonidazole in the management of malignant astrocytoma. A preliminary report. *Cancer*. 1985;56:758-760. doi:10.1016/j.ejca.2015.07.032

20. Shin KH, Muller PJ, Geggie PHS. Superfractionation Radiation-Therapy in the Treatment of Malignant Astrocytoma. *Cancer*. 1983;52:2040-2043. doi:Doi 10.1016/0360-3016(81)90528-9

21. Tamura K, Aoyagi M, Wakimoto H, et al. Accumulation of CD133-positive glioma cells after high-dose irradiation by gamma knife surgery plus external beam radiation: Clinical article. *Journal of Neurosurgery*. 2010;113(2):310-318. doi:10.3171/2010.2.JNS091607

22. Tamura K, Aoyagi M, Ando N, et al. Expansion of CD133-positive glioma cells in recurrent de novo glioblastomas after radiotherapy and chemotherapy. *Journal of Neurosurgery*. 2013;119(5):1145-1155. doi:10.3171/2013.7.JNS122417

23. Pallini R, Ricci-Vitiani L, Montano N, et al. Expression of the stem cell marker CD133 in recurrent glioblastoma and its value for prognosis. *Cancer*. 2011;117(1):162-174. doi:10.1002/cncr.25581

24. Singh SK, Clarke ID, Terasaki M, et al. Identification of a cancer stem cell in human brain tumors. *Cancer Research*. 2003;63(18):5821-5828.

25. Singh SK, Hawkins C, Clarke ID, et al. Identification of human brain tumour initiating cells. *Nature*. 2004;432(7015):396-401. doi:10.1038/nature03128

26. Bao S, Wu Q, McLendon RE, et al. Glioma stem cells promote radioresistance by preferential activation of the DNA damage response. *Nature*. 2006;444(7120):756-760. doi:10.1038/nature05236

27. Zeppernick F, Ahmadi R, Campos B, et al. Stem Cell Marker CD133 Affects Clinical Outcome in Glioma Patients. *Clinical Cancer Research*. 2008;14(1):123-130. doi:10.1158/1078-0432.CCR-07-0932

28. Minata M, Audia A, Shi J, et al. Phenotypic Plasticity of Invasive Edge Glioma Stem-like Cells in Response to Ionizing Radiation. *Cell Reports*. 2019;26(7):1893-1905.e7. doi:10.1016/j.celrep.2019.01.076

29. Contopoulos-Ioannidis DG, Ntzani EE, Ioannidis JPA. Translation of highly promising basic science research into clinical applications. *American Journal of Medicine*. 2003;114(6):477-484. doi:10.1016/S0002-9343(03)00013-5

30. Kimmelman J, Federico C. Consider drug efficacy before first-in-human trials. *Nature*. 2017;542(7639):25-27. doi:10.1038/542025a

31. Tsien C, Pugh S, Dicker AP, et al. Randomized Phase II Trial of Re-Irradiation and Concurrent Bevacizumab versus Bevacizumab Alone as Treatment for Recurrent Glioblastoma (NRG Oncology/RTOG 1205): Initial Outcomes and RT Plan Quality Report. *International Journal of Radiation Oncology Biology Physics*. 2019;105(1):S78.

32. Osuka S, Zhu D, Zhang Z, et al. N-cadherin upregulation mediates adaptive radioresistance in glioblastoma. *Journal of Clinical Investigation*. 2021;131(6). doi:10.1172/JCI136098

33. Volovetz J, Berezovsky AD, Alban T, et al. Identifying conserved molecular targets required for cell migration of glioblastoma cancer stem cells. *Cell Death and Disease*. 2020;11(2). doi:10.1038/s41419-020-2342-2

34. Sato A, Sakurada K, Kumabe T, et al. Association of stem cell marker CD133 expression with dissemination of glioblastomas. *Neurosurgical Review*. 2010;33(2):175-184. doi:10.1007/s10143-010-0239-8

35. Shibahara I, Sonoda Y, Saito R, et al. The expression status of CD133 is associated with the pattern and timing of primary glioblastoma recurrence. *Neuro-Oncology*. 2013;15(9):1151-1159. doi:10.1093/neuonc/not066

36. Yamaki T, Shibahra I, Matsuda K ichiro, et al. Relationships between recurrence patterns and subventricular zone involvement or CD133 expression in glioblastoma. *Journal of Neuro-Oncology*. 2020;146(3):489-499. doi:10.1007/s11060-019-03381-y

37. Keime-Guibert F, Chinot O, Taillandier L, et al. Radiotherapy for Glioblastoma in the Elderly. *New England Journal of Medicine*. 2007;356(15):1527-1535. doi:10.1056/NEJMoa065901

38. Malmström A, Grønberg BH, Marosi C, et al. Temozolomide versus standard 6-week radiotherapy versus hypofractionated radiotherapy in patients older than 60 years with glioblastoma: The Nordic randomised, phase 3 trial. *The Lancet Oncology*. 2012;13(9):916-926. doi:10.1016/S1470-2045(12)70265-6

39. Roa W, Brasher PMA, Bauman G, et al. Abbreviated Course of Radiation Therapy in Older Patients With Glioblastoma Multiforme: A Prospective Randomized Clinical Trial. *Journal of Clinical Oncology*. 2004;22(9):1583-1588. doi:10.1200/JCO.2004.06.082

40. Lemasson B, Galban CJ, Boes JL, et al. Diffusion-weighted MRI as a biomarker for tumor radiation treatment response heterogeneity: a comparative study of whole-volume histogram analysis versus voxel-based functional diffusion map analysis. *Translational Oncology*. 2013;6(5):554-561. doi:10.1593/tlo.13532

41. Bleehen NM, Stenning SP. A medical research council trial of two radiotherapy doses in the treatment of grades 3 and 4 astrocytoma. *British Journal of Cancer*. 1991;64(4):769-774. doi:10.1038/bjc.1991.396

42. Stupp R, Hegi ME, Mason WP, et al. Effects of radiotherapy with concomitant and adjuvant temozolomide versus radiotherapy alone on survival in glioblastoma in a randomised phase III study: 5-year analysis of the EORTC-NCIC trial. *The Lancet Oncology*. 2009;10(5):459-466. doi:10.1016/S1470-2045(09)70025-7

43. Perry JR, Laperriere N, O’Callaghan CJ, et al. Short-Course Radiation plus Temozolomide in Elderly Patients with Glioblastoma. *New England Journal of Medicine*. 2017;376(11):1027-1037. doi:10.1056/NEJMoa1611977

44. Athanassiou H, Synodinou M, Maragoudakis E, et al. Randomized phase II study of temozolomide and radiotherapy compared with radiotherapy alone in newly diagnosed glioblastoma multiforme. *Journal of Clinical Oncology*. 2005;23(10):2372-2377. doi:10.1200/JCO.2005.00.331

45. Wick W, Platten M, Meisner C, et al. Temozolomide chemotherapy alone versus radiotherapy alone for malignant astrocytoma in the elderly: The NOA-08 randomised, phase 3 trial. *The Lancet Oncology*. 2012;13(7):707-715. doi:10.1016/S1470-2045(12)70164-X

46. Pitter KL, Tamagno I, Alikhanyan K, et al. Corticosteroids compromise survival in glioblastoma. *Brain*. 2016;139(5):1458-1471. doi:10.1093/brain/aww046

47. Wirsching HG, Tabatabai G, Roelcke U, et al. Bevacizumab plus hypofractionated radiotherapy versus radiotherapy alone in elderly patients with glioblastoma: The randomized, open-label, phase II ARTE trial. *Annals of Oncology*. 2018;29(6):1423-1430. doi:10.1093/annonc/mdy120

48. Baxter PA, Su JM, Onar-Thomas A, et al. A phase I/II study of veliparib (ABT-888) with radiation and temozolomide in newly diagnosed diffuse pontine glioma: A Pediatric Brain Tumor Consortium study. *Neuro-Oncology*. 2020;22(6):875-885. doi:10.1093/neuonc/noaa016

49. Sim HW, Mcdonald KL, Lwin Z, et al. A randomized phase II trial of veliparib, radiotherapy, and temozolomide in patients with unmethylated MGMT glioblastoma: The VERTU study. *Neuro-Oncology*. 2021;23(10):1736-1749. doi:10.1093/neuonc/noab111
